# Poverty, Genocide, and Superbugs: A Carbapenem-Resistant Wound Infection in Rural Guatemala

**DOI:** 10.4269/ajtmh.18-0260

**Published:** 2018-09

**Authors:** David Flood

**Affiliations:** 1 University of Minnesota, Minneapolis, Minnesota;; 2 Wuqu’ Kawoq, Santiago Sacatepéquez, Sacatepéquez, Guatemala

My last memory of Esteban haunts me, but I choose to remember most the day he showed up at a rural Guatemalan clinic in November 2013. A slender indigenous Maya man in his early forties who had remarkably severe type 2 diabetes, Esteban had been missing from the clinic for nearly 2 years.

Observing the surprised faces of the clinic staff upon arrival, he explained that he had migrated to the United States from his village to look for work. However, he had recently decided to return to Guatemala because obtaining diabetes treatment in the United States was too difficult and expensive. He was back in the clinic to resume his free care. “I don’t know how people with diabetes make ends meet in your country!” he told me as he flashed a mischievous grin.

A few months earlier, I had begun working with a nongovernmental health organization serving indigenous communities in Guatemala. One of its flagship programs delivered medical care to about 150 adults with type 2 diabetes, most of whom were indigenous Mayas. Many patients, like Esteban, spoke one of Guatemala’s many Mayan languages, lived in isolated rural areas, and had relatively limited access to health services. The organization’s diabetes programming was compelling to me as it was of high quality, provided at no cost, and primarily managed by local Maya physicians and nurses.

On his return to the organization’s clinic, Esteban reported persistent hyperglycemic symptoms, his hemoglobin A1C was greater than 13, and he had not been injecting insulin as he had been unable to afford it. The clinic staff re-prescribed him NPH insulin, dispensed lancets and glucose monitoring supplies, and titrated his insulin dose over numerous subsequent phone calls and visits.

He did well. Over the next couple of years, Esteban’s glucose was well controlled with most of his hemoglobin A1C measurements below 7.5. It appeared as though he had avoided the debilitating complications that afflicted many other diabetes patients in the clinic such as amputations, renal failure, and blindness.

I ran across Esteban a few times in the clinic during that period of time. He got a kick out of talking with me, always giving me that same mischievous grin as when we had first met. He seemed to think that we had a special transnational connection and that my presence as an American was hilarious—even absurd. He had chosen to return home to his rural Guatemala village from the United States, and who does he immediately find at the clinic? A gringo!

In August 2015, one of the diabetes nurses informed me that Esteban had developed a severe foot infection requiring admission to a local hospital. A few weeks earlier, he had sustained a minor trauma to his foot, and, although he had thought nothing of it at the time, he soon developed an infected diabetic ulcer with pain, swelling, and purulent discharge. In the hospital, his infection was thought to be severe enough that he was at risk of needing an amputation. Fortunately, his ulcer improved with appropriate management, but, during the hospitalization, he also developed a pressure ulcer over his hip from a lack of repositioning. In addition, Esteban’s family spent down a large portion of their assets during the hospitalization, as they were required to purchase essential medicines and supplies that were unavailable at the public hospital.

I saw Esteban for the last time on a sunny, warm day in December 2015. He came to the clinic alongside his wife, Magda, a petite woman dressed in a traditional Maya blouse and skirt. Because of his continued immobility and the lack of rehabilitation facilities or home nursing care in Guatemala, Esteban’s ulcer had worsened and had become infected. On examination, the clinic’s diabetes nurse and I observed a massive necrotizing wound over Esteban’s hip draining a large quantity of pus. His wife showed us the results of a wound culture revealing the growth of a *Klebsiella* species resistant to all antimicrobials tested, including carbapenems.

The diabetes nurse immediately contacted her clinical supervisor, and together they agreed that only immediate rehospitalization for debridement and intravenous antibiotics could possibly save Esteban’s life.

Hearing this, Esteban snorted in disgust at the prospect of returning to the hospital, which he viewed as provoking this secondary complication, treating him poorly as an indigenous patient, and extracting scarce family resources. “I’m not going back to the hospital,” he declared. “Better that I die at home.”

Approximately a month later, Esteban died at home. I was later informed that his family had made a last-ditch effort to treat his infection by purchasing various antibiotics at a local private clinic, including levofloxacin, although his infection was resistant to fluoroquinolones. His wife became a single mother to several young children.

How did this happen? Where did he acquire such a highly resistant infection? Why did he die so young?

I continue to reflect upon these questions now, years after Esteban’s death.

Esteban was born into poverty and had the bad luck of developing diabetes at an early age. Because of his family’s geographic isolation and lack of resources, he was unable to control his diabetes early in its disease course. As a result, he was more susceptible to developing complications such as infected ulcers at an early age. He may have acquired the carbapenem-resistant organism during his hospitalization, as resistant *Klebsiella* is notorious for nosocomial transmission and has been reported widely in hospitals in Latin America. Or the bug may have developed or have been transmitted to Esteban from the community, as a lack of pharmaceutical regulation in Guatemala facilitates antimicrobial resistance as antibiotics can be freely purchased and dispensed without prescription.

The aforementioned explanatory model for Esteban’s case is not quite incorrect, but it is incomplete.

A more nuanced interpretation considers the social, political, and historical factors of Esteban’s case. For example, the centuries-long history of colonization and oppression in Guatemala has led Maya people like Esteban to suffer from intractable intergenerational poverty and discrimination. The Guatemalan government has a low expenditure on health, which contributes to inadequate public health infrastructure and limited pharmaceutical regulation. The inequitable health-care system in the United States caused Esteban to face significant barriers to receiving diabetes care. Finally, the 1954 Central Intelligence Agency-supported coup in Guatemala and resulting civil war from 1960 to 1996 profoundly impacted Esteban.

During the war, the United States gave direct and indirect support to Guatemalan military forces in a campaign against rural, predominantly indigenous groups that killed more than 200,000 people. The Guatemalan truth commission concluded that the violence constituted an act of genocide against Maya communities.

Esteban’s small village of just over 100 houses was the site of one of the region’s most brutal massacres. Tucked in the truth commission’s 1999 report, “Memory of Silence,” are descriptions of two Guatemalan Army assaults against the village in 1981 and 1982. In the first attack, army soldiers tortured and executed more than a dozen villagers, set fire to the homes, and killed the village’s animals. In the second attack, the army bombed Esteban’s village with airplanes and helicopters, resulting in more than 60 deaths, including many women and children. Army soldiers subsequently arrived in the village, robbed the villagers’ remaining goods and livestock, and forced survivors to dig a mass grave for the victims.

Esteban was approximately 10 years old at the time of these events. We never talked about it, but I have often wondered what his experience was during the attacks, which family members he lost, and how the violence impacted him as he grew up. In a village as small as Esteban’s, it is likely that many of his family members were killed.

The war certainly contributed to his family’s poverty and to his poor diabetes control over the years. It also may have led to his decision to migrate to the United States where he was unable to access diabetes care. In this context, it does not seem far-fetched to link his highly resistant infection to a genocidal civil war.

One of the clinic’s diabetes nurses, who lives near Esteban’s village, tells me that she sees Esteban’s widow, Magda, every few months in the town market. They greet each other, and it seems like things are going okay. Magda runs a small shop from her home to make ends meet.

When she comes to the market, Magda carries on her back a healthy young infant wrapped tightly in the traditional Maya style within a bundle of weavings. The nurse informed me that Magda was pregnant when Esteban died and that this infant was born a few months after his death.

The World Health Organization lists carbapenem-resistant bacteria as the highest priority pathogens in terms of their threat to human health. Esteban’s case illustrates that these highly resistant bugs develop and are transmitted within a social and historical context of poverty, violence, and discrimination. In this way, Esteban’s infection reflects the intimate suffering of individuals, families, and communities—not only in the present, but also in the past and future.

